# Selectins in Biology and Human Disease: Opportunity in E-selectin Antagonism

**DOI:** 10.7759/cureus.61996

**Published:** 2024-06-09

**Authors:** John M Peterson, Theodore A Smith, Edwin P Rock, John L Magnani

**Affiliations:** 1 Research, GlycoMimetics, Inc., Rockville, USA; 2 Development, GlycoMimetics, Inc., Rockville, USA; 3 Research and Development, GlycoTech Corporation, Rockville, USA

**Keywords:** thrombosis, acute lung injury, vaso-occlusion, sickle cell disease, antagonism, leukocyte activation, inflammation, adhesion, selectin

## Abstract

Selectins are cell adhesion proteins discovered in the 1980s. As C-type lectins, selectins contain an essential calcium ion in the ligand-binding pocket and recognize the isomeric tetrasaccharides sialyl Lewis^x^ (sLe^x^) and sialyl Lewis^a^ (sLe^a^). Three selectins, E-selectin, P-selectin, and L-selectin, play distinct, complementary roles in inflammation, hematopoiesis, and tumor biology. They have been implicated in the pathology of diverse inflammatory disorders, and several selectin antagonists have been tested clinically. E-selectin plays a unique role in leukocyte activation, making it an attractive target for intervention, for example, in sickle cell disease (SCD). This review summarizes selectin biology and pathology, structure and ligand binding, and selectin antagonists that have reached clinical testing with an emphasis on E-selectin.

## Introduction and background

All cell surfaces in nature are coated with glycans as components of glycoproteins and glycolipids. These complex carbohydrates present structural information used for recognition, cellular adhesion, and signaling. Receptors that bind these structurally diverse ligands are known as carbohydrate-binding proteins or lectins, a term derived from the Latin "legere," meaning to read or choose [1]. Lectins are prevalent throughout phylogeny, including in microbial, plant, and animal life [2].

C-type lectins require calcium for binding via a Ca^++^-dependent carbohydrate-recognition domain (CRD). Among C-type lectins, the selectin family of cell adherence receptors includes P-, E-, and L-selectins that were originally identified on platelets, endothelial cells, and lymphocytes, respectively (Table 1) [3-5]. These carbohydrate-binding adhesion proteins mediate inflammation, leukocyte trafficking, and bone marrow homeostasis [6,7]. Correspondingly, selectins have been identified as pathogenic drivers of diverse inflammatory and malignant disease states. E-selectin in particular appears to be a promising target for the treatment of inflammatory disorders and cancers.

**Table 1:** Selectin family proteins LFA-1: leukocyte function-associated antigen-1, sLe^x^: sialyl Lewis^x^ References: [3-6]

	Location	Physiology	Structure and function
P-selectin	Expressed on activated endothelium and activated platelets	Pre-synthesized and stored in platelet $\alpha$*-*granules and endothelial cell Weibel-Palade bodies, expressed within minutes of inflammatory activation	Binds sLe^x^ on sulfated glycoproteins, captures circulating leukocytes and platelets, allows for rolling on activated endothelium
E-selectin	Constitutively expressed in bone marrow vasculature, inducibly expressed on activated endothelium	Supports hematopoietic stem cell proliferation, de novo synthesis for hours after inflammatory activation, induces LFA-1 activation and firm adhesion to endothelial cells	Binds sLe^x^ on L-selectin, other glycoproteins, and glycolipids, captures circulating leukocytes, allows for rolling on activated endothelium
L-selectin	Constitutively expressed on leukocytes	Enables lymphocyte homing to lymph nodes, enables leukocyte clustering at sites of inflammation	Displays sLe^x^ that binds to E-selectin and leads to LFA-1 activation

This review describes selectins, the rationale for targeting them therapeutically, and progress in advancing such therapies. Selectins and the structural features of their interactions with selectin ligands will be summarized. Reflection on selectin biology then informs the rationale for E-selectin inhibition and identification of promising target diseases. Subsequent description of selectin crystal structures and ligand binding details enables consideration of pathways to design specific selectin antagonists. Finally, clinical trials to date of specific selectin antagonists will be summarized.

## Review

Selectins and their ligands

**Figure 1:**
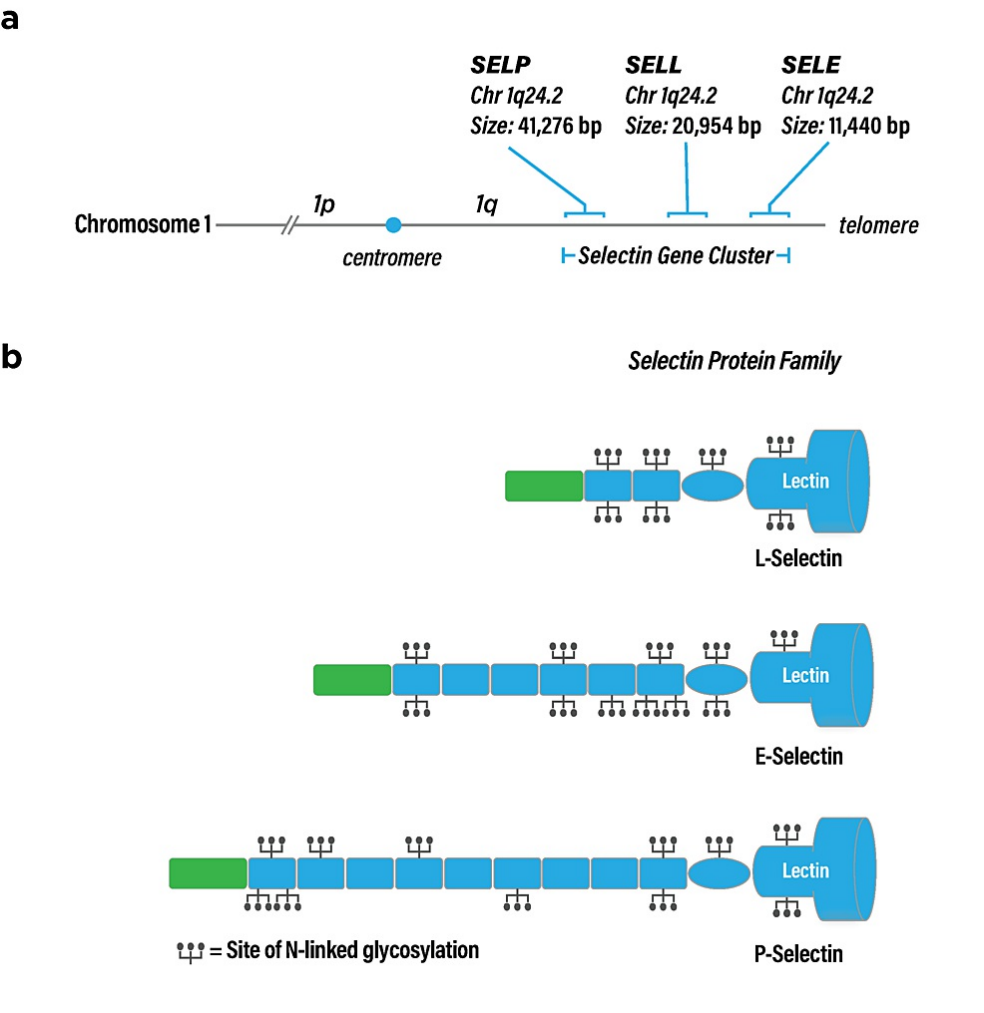
Selectin genes and protein domain structure (a) Selectin gene cluster on chromosome 1q. (b) Selectin protein structure. The N-terminal CRD is labeled "lectin" at the right side of the figure, followed by an EGF domain (oval) and varying numbers of SCR domains (rectangles). Intracellular domains are represented by green rectangles, and glycosylation sites are represented by tridents. CRD: carbohydrate-recognition domain, EGF: epidermal growth factor, SCR: short consensus repeat Image credits: Dr. Drew Provan using Adobe Illustrator

The selectin family includes three members that appear to have evolved by duplication of a common ancestral gene (Figure 1a). Correspondingly, three selectin genes reside within a ~150 kilobase pair gene locus at chromosome 1q24 (Figure 1a) [8]. Structurally, selectins comprise an N-terminal calcium-dependent carbohydrate recognition domain (CRD), followed by an epidermal growth factor-like (EGF) domain, a variable number of short consensus repeat (SCR) domains, a single membrane-spanning domain, and a C-terminal intracellular domain (Figure 1b). P-selectin has nine SCR domains, while E-selectin is shorter with six domains, and L-selectin is the shortest with two [6]. SCR domains reportedly modulate binding ligand potency with a greater number of SCRs leading to more potent binding [9]. Reflecting the modular nature of selectins, each protein domain is encoded by a separate exon, except for the cytoplasmic tails, which are each encoded by two exons [10-12].

**Figure 2:**
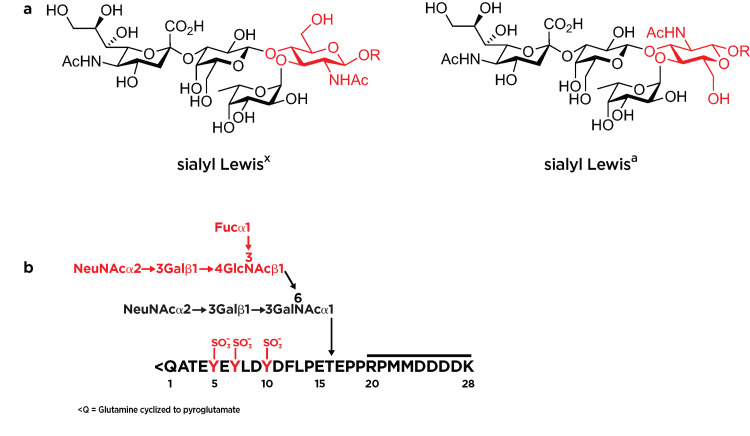
Selectin binding ligand structures (a) Structures of sialyl Lewis^x^ and sialyl Lewis^a^​​​​​​​: the GlcNAc residue is highlighted in red to indicate the isomeric nature of the structure. (b) Structure of synthetic P-selectin ligand SGP-3, comprising the terminal 19 amino acids of PSGL-1, including three sulfated tyrosine residues (sY5, sY7, and sY10) (highlighted in red), an O-linked saccharide at T16 containing a sialyl Lewis^x^​​​​​​​ (highlighted in red) moiety, and an enterokinase cleavage sequence from R20 to K28 [16]. PSGL-1: P-selectin glycoprotein ligand-1 Image credits: Dr. Drew Provan using Adobe Illustrator

Selectins bind to isomeric terminal tetrasaccharide epitopes sialyl Lewis^x^ (sLe^x^) and sialyl Lewis^a^ (sLe^a^) (Figure 2a) [13-15]. In addition to these carbohydrate motifs, the binding of P-selectin requires the presence of nearby sulfated tyrosine residues, as in the P-selectin ligand SGP-3 (Figure 2b) [14-16]. L-selectin can also recognize sulfated analogs of sLe^x^ [17]. These binding motifs have been identified on multiple glycoproteins, including P-selectin glycoprotein ligand-1 (PSGL-1), E-selectin ligand-1 (ESL-1), and the CD44 glycoform hematopoietic cell E-/L-selectin ligand (HCELL) [18,19]. Human L-selectin is unique among selectins in itself displaying the sLe^x^ epitope, so it can also act as a ligand for E-selectin, although not for P-selectin or L-selectin [19-21].

Selectin biology

Selectins Lead to Inflammatory Cell Binding, Leukocyte Clustering, and Firm Adhesion

**Figure 3:**
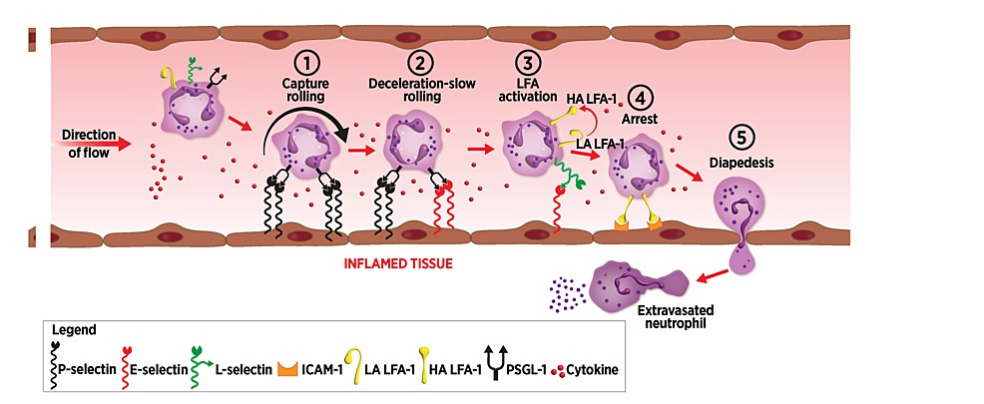
Inflammatory cascade leading to cell migration (1) Inflammation activates local endothelium to express P-selectin that initiates tethering and rolling of circulating leukocytes along the endothelial wall. (2) Inflammatory cytokines induce de novo synthesis and surface expression of E-selectin that also captures circulating neutrophils. (3) E-selectin binds to sLe^x^ residues expressed by L-selectin on captured neutrophils, inducing conformational changes of LFA-1 from an LA to an intermediate affinity, then an HA state. (4) LFA-1 in its HA conformation binds to ICAM-1 expressed by endothelial cells, leading to firm arrest. (5) Following firm arrest, leukocytes extravasate into inflamed tissue by the process of diapedesis. sLe^x^: sialyl Lewis^x^, LFA-1: leukocyte function-associated antigen-1, LA: low affinity, HA: high affinity, ICAM-1: intercellular adhesion molecule-1, PSGL-1: P-selectin glycoprotein ligand-1 Image credits: Dr. Drew Provan using Adobe Illustrator

As shown in Figure 3, selectins act temporally in concert during inflammation. In areas of tissue inflammation, cytokines such as tumor necrosis factor-$\alpha$ (TNF-$\alpha$) and interleukin-1 (IL-1) are released, activating local endothelium. P-selectin is released from endothelial Weibel-Palade bodies within minutes of activation, expressed on the cell surface, and subsequently shed or internalized within hours [22-24]. Binding ligands for P-selectin, prominently including PSGL-1, are clustered at the tips of circulating neutrophil microvilli. Cells captured from circulation roll along venular endothelium via selectin/PSGL-1 bond formation at the leading edge of the rolling neutrophil with corresponding bond breakage at the trailing edge.

E-selectin expression begins in the hours after an inflammatory stimulus after de novo transcription, translation, and surface display. Shedding or internalization occurs roughly 24 hours later [24]. Like P-selectin, E-selectin mediates the capture and rolling of circulating neutrophils. These arrested leukocytes contribute to the formation of thrombi and vascular occlusions.

Neutrophils and monocytes are also captured by L-selectin binding to activated PSGL-1 expressed on the endothelial surface [25,26]. L-selectin on the rolling leukocyte amplifies inflammatory responses by binding to ligands, mainly PSGL-1, on circulating neutrophils, leading to leukocyte clustering and secondary capture [27-29].

In addition to capture and rolling, E-selectin and L-selectin interact to trigger slow rolling, arrest, and firm adhesion of rolling leukocytes, followed by diapedesis. E-selectin binding to sLe^x^ on neutrophil L-selectin induces inflammasome activation and release of myeloid-related protein 8/14 (MRP8/14) (also known as SA100A8/9 or calprotectin) that binds toll-like receptor 4 (TLR4). E-selectin binding induces a conformational change in $\beta$-2 integrin leukocyte function-associated antigen-1 (LFA-1) from a low to a medium affinity state [30,31]. This change mediates slow rolling from increased LFA-1 binding affinity to intercellular adhesion molecule-1 (ICAM-1). Subsequent LFA-1 conversion to a high-affinity state induces arrest and firm adhesion [31]. Diapedesis follows as cells migrate from endothelium into inflamed tissue.

In addition to its role in innate immune responses, L-selectin functions as a mediator of leukocyte trafficking between blood and lymph [32]. High endothelial venule cells in lymph nodes express sulfated glycoproteins that bind to L-selectin on circulating leukocytes, enabling lymphocyte transfer from blood to lymph nodes [33]. This transfer is vital for immune surveillance by enabling circulating lymphocytes to reach antigen-presenting cells in lymphatic tissue.

Genetic Deletions of Selectin Genes Further Inform Their Biology

Selectins have been studied in gene knockout (KO) mice [34]. L-selectin KO mice show reduced migration of neutrophils, lymphocytes, and monocytes in several models of inflammation, as well as a dramatic reduction of lymphocyte migration into lymph nodes [33,35]. Similarly, P-selectin KO mice display no leukocyte rolling during the first minutes after exteriorization of mesenteric venules, confirming a role for P-selectin in early leukocyte capture in mice [36,37]. Correspondingly, P-selectin KO mice also show prolonged bleeding times versus wild-type littermates, confirming a role for P-selectin in mouse hemostasis [38]. Conversely, E-selectin KO mice show little difference in peritoneal neutrophil migration after a thioglycolate challenge, compared to wild-type littermates [39]. However, E-selectin KO mice display markedly fewer arrested leukocytes after TNF-$\alpha$ stimulation, consistent with E-selectin's role in cell arrest due to integrin activation [40]. E-selectin KO mice also show reduced proliferation of hematopoietic stem cells and enhanced survival of these cells after treatment with cytotoxic chemotherapy or ionizing radiation [41].

Mice with gene deletions of both E-selectin and P-selectin show marked decreases in neutrophil rolling on endothelium after TNF-$\alpha$ challenge, compared to wild-type littermates. Correspondingly, these double KO mice are also susceptible to infection and develop ulcerative dermatitis [42]. Mice with deletions of all three selectins show marked decreases in leukocyte rolling after TNF-$\alpha$ activation yet curiously do not develop the skin lesions seen in E-selectin/P-selectin double knockouts [43].

Extrapolating selectin function from mice to humans warrants caution as important differences distinguish murine and human immune systems [44]. For example, neutrophils comprise 50%-70% of total white blood cells (WBCs) in humans versus 10%-25% in mice. Conversely, human lymphocytes account for 30%-50% of white blood cells in humans versus 75%-90% in mice [44-46]. Importantly, TNF-$\alpha$ and lipopolysaccharide induce P-selectin gene transcription in mice, but not in humans, and P-selectin in humans appears to contribute only minimally to leukocyte capture [47,48]. Finally, murine neutrophils lack the fucosyltransferase enzyme needed to synthesize sLe^x^ [49,50]. Thus, L-selectin on human neutrophils serves as an E-selectin ligand, whereas murine L-selectin does not [51].

Soluble Selectins Are Markers of Neutrophil, Platelet, and Endothelial Inflammation

While selectins are membrane-bound, soluble forms of each are found in plasma, either as isolated proteins or as components of microparticles. Soluble selectins are generated from the proteolytic cleavage of membrane-bound selectins and contain only extracellular domains. Detectable in blood even in the absence of overt inflammation, circulating soluble selectins may influence inflammation, although their precise functions remain incompletely described. Notably, soluble selectin levels are adversely prognostic in multiple disease states [52-55].

Soluble L-selectin is formed from L-selectin cleavage by the zinc-dependent protease a disintegrin and metalloproteinase 17 (ADAM17); other proteases may also be involved [56]. Within minutes of endothelial activation and neutrophil adhesion, soluble L-selectin can be measured in supernatants of in vitro adhesion assays [57,58]. Thus, soluble L-selectin serves as a marker of neutrophil activation. Mechanisms for E-selectin and P-selectin shedding are less well understood, although their appearance aligns with the kinetics of their roles in inflammation. Platelets are a primary source for circulating soluble P-selectin, which is shed from the platelet surface within minutes of activation [59]. As such, soluble P-selectin serves as a marker for platelet activation. E-selectin is shed 24 hours after the activation of human umbilical vein endothelial cells, but the responsible protease(s) remains unknown. Soluble E-selectin serves as a marker for endothelial inflammatory activation [60].

Targeting Selectins to Treat Inflammatory Disorders

As therapeutic targets, the unique roles of each selectin family member inform their relative attractiveness for inhibitory targeting. Due to its involvement in capture and secondary capture, L-selectin targeting would be expected to have anti-inflammatory and/or anti-thrombotic effects. However, its actions go beyond local inflammation to an important role in lymphocyte trafficking to lymph nodes [61]. As such, L-selectin inhibition might impair adaptive immune responses and has not been widely pursued as a therapeutic target. With its role in early mouse yet not human leukocyte capture and rolling, P-selectin has also been an attractive target. However, its endothelial expression is relatively brief, so its antagonism might be better suited for prophylaxis settings, rather than the management of ongoing acute inflammatory events.

E-selectin is unique among selectins in mediating not only leukocyte tethering and rolling but also integrin activation that in turn underlies slow rolling, arrest, and firm adhesion. Also, inflammatory cytokines, such as TNF-$\alpha$, induce the synthesis of E-selectin but decrease P-selectin mRNA in humans [48]. Finally, the E-selectin expression duration exceeds that of P-selectin. Appropriately timed E-selectin blockade (before integrin-mediated firm adhesion) may be a practical target for the treatment of inflammatory disorders. Therefore, this review focuses primarily on E-selectin correlations with disease and E-selectin antagonists in clinical trials. For completeness, P-selectin antagonists that have reached clinical testing are also covered.

E-selectin in pathology

As an inflammatory cascade mediator, E-selectin participates in diverse inflammatory processes. Also, because soluble E-selectin serves as a biomarker of inflammatory endothelial activation, disorders featuring elevated soluble E-selectin reveal associated pathology. Correspondingly, numerous studies find that soluble E-selectin levels correlate with disease and/or disease severity (Table 2). A common mechanism among these conditions is endothelial activation, followed by inflammatory cell adhesion, extravasation, and tissue pathology.

**Table 2:** E-selectin and its ligand in disease states sE: soluble E, sP: soluble P, AML: acute myeloid leukemia, ARDS: acute respiratory distress syndrome, CAD: coronary artery disease, HDL: high-density lipoprotein, SCD: sickle cell disease, TIA: transient ischemic attack, ICU: intensive care unit

Acute lung injury/critical care	Reference
Plasma sE-selectin and sP-selectin are elevated among ARDS patients	[62]
Patients requiring ICU care had elevated sE-selectin levels	[63]
Serum sE-selectin is higher in idiopathic pulmonary fibrosis than controls	[64]
Inflammatory diseases	
sE-selectin is elevated in sera of patients with atopic dermatitis and psoriasis	[65]
Significantly increased sE-selectin levels were found in psoriatic patients	[66,67]
sE-selectin is increased in inflammatory bowel disease	[68]
sE-selectin levels are associated with activity and outcome of rheumatoid arthritis	[69]
Median sE-selectin in sarcoidosis was elevated versus controls	[70]
Serum sE-selectin was higher in active than inactive sarcoidosis and controls	[71]
High sE-selectin was observed in patients with localized scleroderma	[72]
Significantly elevated sE-selectin levels were found in patients with lupus	[73]
Metabolic diseases	
Types 1 and 2 diabetes mellitus is associated with elevated sE-selectin	[74,75]
sE-selectin is elevated in hyperlipidemic patients versus normolipemic controls	[76,77]
sE-selectin is elevated in patients with elevated triglyceride and reduced HDL levels	[78]
Non-alcoholic fatty liver shows higher hepatic E-selectin and plasma sE-selectin	[79]
Plasma sE-selectin levels were elevated in obese subjects	[80]
Oncologic diseases	
E-selectin ligand expression on acute myeloid leukemia cells is higher in patients with relapsed than newly diagnosed disease, high-risk cytogenetics, and secondary AML	[81]
Plasma sE-selectin is elevated in chronic myeloproliferative disorders	[82]
Serum sE-selectin with invasive breast cancer is higher than in healthy controls	[83]
sE-selectin is significantly elevated in patients with metastatic breast cancer	[84]
Sickle cell disease	
Plasma levels of sE-selectin are associated with retinopathy in SCD	[85]
sE-selectin is higher in sickle cell patients than controls and increases during vaso-occlusive episodes	[86]
sE-selectin levels correlate with mortality in sickle cell patients	[87]
Thromboembolic diseases	
sE-selectin is elevated in hypertensive patients	[88,89]
High sE-selectin is observed in patients with myocardial infarction	[90]
Increased plasma sE-selectin is observed in acute myocardial infarction	[91]
sE-selectin is significantly elevated in patients with coronary heart disease	[88]
Elevated sE-selectin is observed in unstable and variant angina	[92,93]
Increased sE-selectin levels are found in patients with ischemic heart disease	[94]
Stroke and TIA showed significantly increased levels of sE-selectin	[95,96]

E-selectin in Acute Lung Injury (ALI) and Acute Respiratory Distress Syndrome (ARDS)

**Figure 4:**
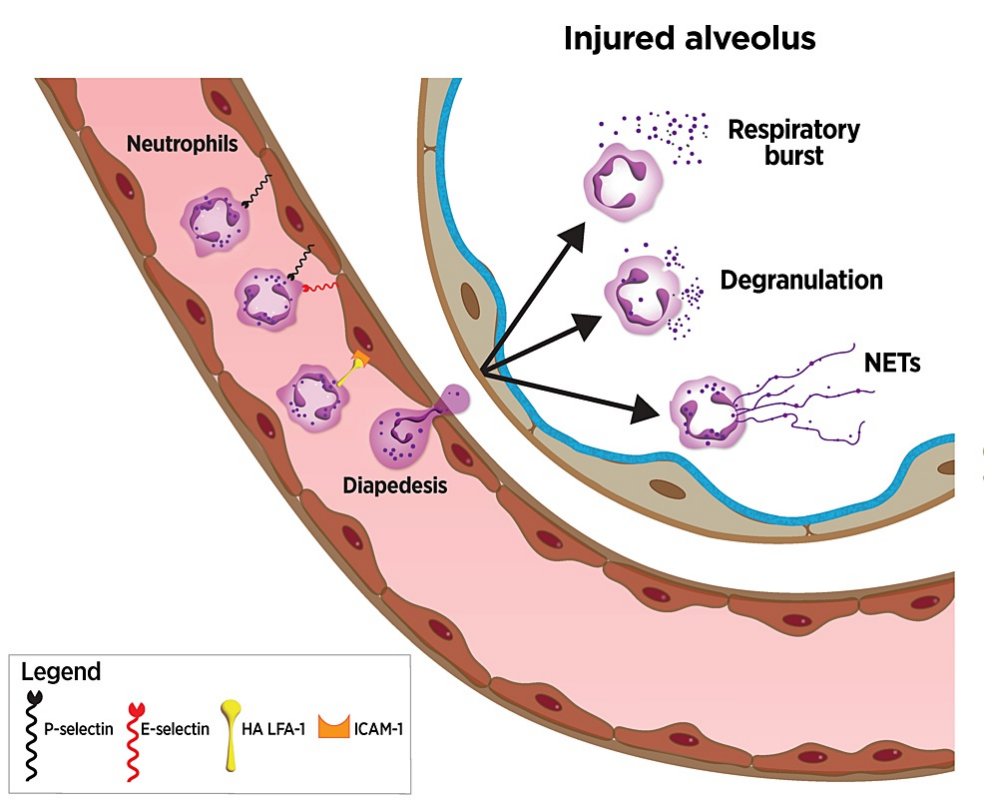
Neutrophil extravasation and acute lung injury or pneumonia In severe lung inflammation, activated endothelial surface selectin expression leads to circulating neutrophil capture, extravasation into alveoli, and activation leading to alveolar fluid accumulation and impaired gas exchange. To eliminate redundancy, the binding cascade here omits PSGL-1 interactions with P-selectin and E-selectin, as well as E-selectin interactions with L-selectin. PSGL-1: P-selectin glycoprotein ligand-1, NETS: neutrophil extracellular traps, HA: high affinity, LFA-1: leukocyte function-associated antigen-1, ICAM-1: intercellular adhesion molecule-1 Image credits: Dr. Drew Provan using Adobe Illustrator

ALI and ARDS are associated with acute respiratory failure, substantial morbidity, and in-hospital mortality of about 41% [97]. Diverse factors triggering ARDS include infection, sepsis, trauma, and aspiration. Across causes, elevated plasma IL-6, IL-8, and TNF-$\alpha$ levels are associated with increased mortality [98]. ALI and ARDS feature acute inflammation that disrupts lung epithelial and endothelial barriers. Neutrophil extravasation into lung tissue precedes degranulation with the release of reactive oxygen species (ROS), neutrophil extracellular traps (NETs), and inflammatory cytokines (Figure 4) [99]. Neutrophil activation promotes alveolar basement membrane destruction, leading to increased permeability of the alveolar-capillary barrier and alveolar accumulation of protein-rich fluid that impairs gas exchange.

Standard of care ARDS therapy includes mechanical ventilation and fluids. Additional supportive care may include corticosteroids, nitric oxide, surfactants, neuromuscular blocking agents, and/or $\beta$-2-adrenergic agonists. Randomized phase 3 trials have not consistently supported clinical benefit, and treatment efficacy is challenged by the rapid progression of this clinical syndrome and advanced stage at presentation. As E-selectin is required for neutrophil extravasation from blood to lung tissue in ARDS, it presents an appealing potential target for therapeutic intervention. Support for therapeutic E-selectin antagonism comes from a rat preclinical model of acute lung injury characterized by neutrophil influx with extensive alveolar-capillary basement membrane disruption, as in ARDS. Intravenous treatment with purified native ligand for E-selectin blocked neutrophil influx and maintained alveolar-capillary barrier integrity [100]. Also, soluble E-selectin (sE-selectin) levels in plasma predict ARDS progression [101]. Likewise, increasing sE-selectin is associated with organ failure in systemic inflammatory response syndrome (SIRS) patients [102,103].

E-selectin in Sickle Cell Vaso-Occlusive Episodes (VOEs)

**Figure 5:**
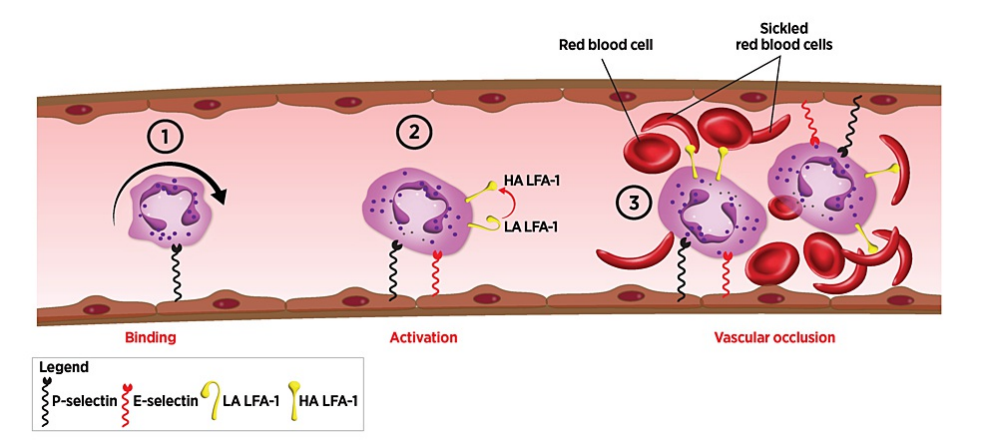
Vaso-occlusion in sickle cell disease (1) Inflammatory triggers, such as hypoxia and hemolysis products, activate endothelium to express P-selectin, which captures circulating leukocytes. (2) E-selectin is expressed following de novo synthesis, capturing more leukocytes. (3) E-selectin binding to sLe^x^ on L-selectin (not shown in the figure), followed by the activation of LFA-1, induces endothelial capture of inflammatory cells, as well as secondary capture of platelets and red blood cells, leading to vascular occlusion. To eliminate redundancy, this binding cascade omits PSGL-1 interactions with P-selectin and E-selectin, as well as E-selectin interactions with L-selectin. sLe^x^: sialyl Lewis^x^, LFA-1: leukocyte function-associated antigen-1, PSGL-1: P-selectin glycoprotein ligand-1, LA: low affinity, HA: high affinity Image credits: Dr. Drew Provan using Adobe Illustrator

Sickle cell disease (SCD) follows the inheritance of a gene mutation that leads to hemoglobin polymerization on deoxygenation, causing erythrocyte membrane damage and the generation of sickle morphology. Endothelial activation caused by blood hemolysis products, low oxygen, and other potential activators induces E-selectin expression and circulating neutrophil capture (Figure 5). Sickle-shaped erythrocytes adhere to and are mechanically entrapped by leukocytes immobilized on venular endothelium. Consequent cytokine secretion and tissue factor release exacerbate endothelial cell activation and E-selectin expression, inducing coagulation and thrombosis, blocking blood flow, and leading to the development of acutely painful vaso-occlusive episodes (VOEs). Chronic tissue ischemia further leads to sequelae including retinopathy, renal failure, osteonecrosis, pulmonary hypertension, stroke, and in some patients, premature death. Given the pathophysiologic contributions of endothelial activation, neutrophil adhesion, and secondary capture of red blood cells (RBCs), selectins have been proposed as targets for the treatment of VOEs [104].

E-selectin and Thrombosis

Venous thromboembolism (VT), including deep vein thrombosis (DVT) and pulmonary embolism (PE), afflicts 900,000 people annually in the United States, causing over 60,000 deaths [105]. Current treatments include direct thrombin inhibitors, vitamin K antagonists, and low-molecular-weight heparin (LMWH). Post-thrombotic syndrome affects up to half of DVT patients with manifestations including leg pain, swelling, skin discoloration, fatigue, and venous ulcers. All current treatments generate a bleeding risk, and none prevents post-thrombotic syndrome, indicating the unmet need for improved therapies.

**Figure 6:**
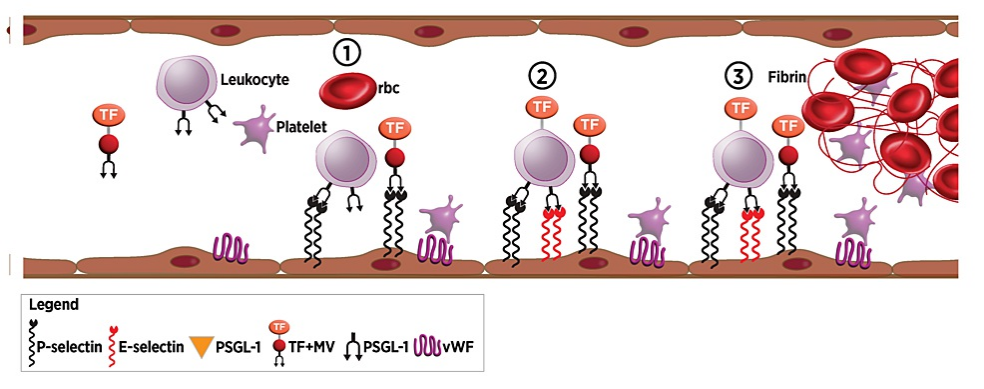
Thrombosis (1) Endothelium is activated via hypoxia or inflammatory triggers leading to the expression of P-selectin, E-selectin, and vWF. These proteins capture circulating platelets, leukocytes, and TF+MV. (2) Activation of bound leukocytes leads to the expression of tissue factors on their surface. Captured platelets also release tissue factor. (3) Tissue factor then activates the clotting cascade, leading to thrombus formation, including rbc. vWF: von Willebrand factor, TF+MV: tissue factor expressing microvesicles, rbc: red blood cells, PSGL-1: P-selectin glycoprotein ligand-1 Image credits: Dr. Drew Provan using Adobe Illustrator

Diverse factors trigger thrombus formation. Endothelial injury from trauma, surgery, or infection can lead to P-selectin and then E-selectin expression, as well as tissue factor release that with factor VIIa leads to fibrin deposition and platelet activation (Figure 6). Hypercoagulability due to autoimmune disorders or genetic deficiencies also raises thrombosis risk with venous stasis inducing a pro-inflammatory endothelial phenotype that leads to neutrophil and monocyte recruitment, as well as fibrin deposition (Figure 6) [106,107]. E-selectin mediates the adhesion of neutrophils and monocytes to damaged endothelium, contributing to thrombus formation. Consequently, E-selectin presents a potential target for strategies to inhibit thrombosis.

Selectin protein structures, ligand binding, and the basis of catch bonds

Effective therapeutic E-selectin targeting relies on a thorough understanding of selectin structural biology. This section describes selectin protein structure similarity, conformational change induced in E-selectin and P-selectin on ligand binding, and the molecular nature of catch bond formation in E-selectin versus P-selectin. These structural features underlie the molecular design of specific select and antagonists.

**Figure 7:**
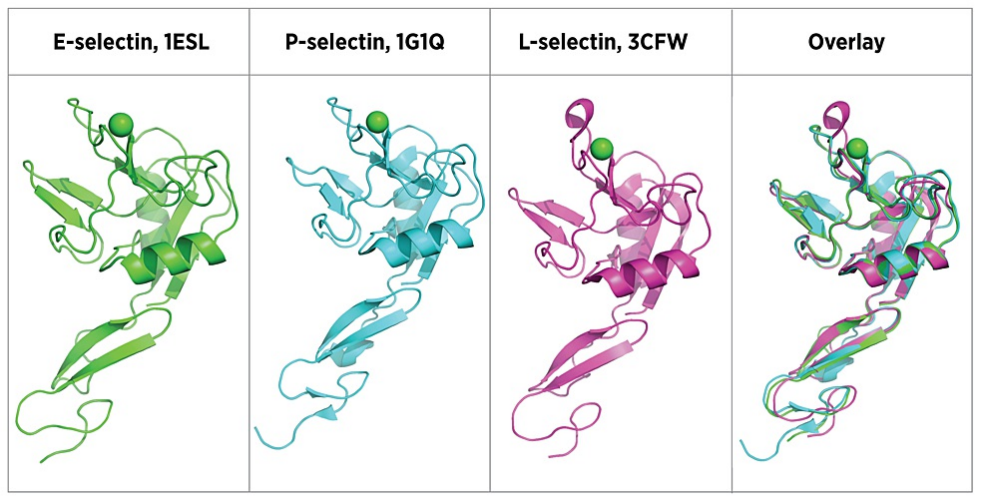
Selectin family X-ray crystal structures Tertiary structures of E-selectin, P-selectin, and L-selectin CRD and EGF-like domains from X-ray crystallography. RCSB Protein Data Bank reference numbers are shown. CRD: carbohydrate-recognition domain, EGF: epidermal growth factor, RCSB: Research Collaboratory for Structural Bioinformatics Image credits: Dr. Drew Provan using Pymol

Numerous selectin crystal structures have been reported [16,108-111]. Overlay of CRD and EGF domain crystal structures of E-selectin, P-selectin, and L-selectin indicate similar, essentially superimposable tertiary structures (Figure 7).

**Figure 8:**
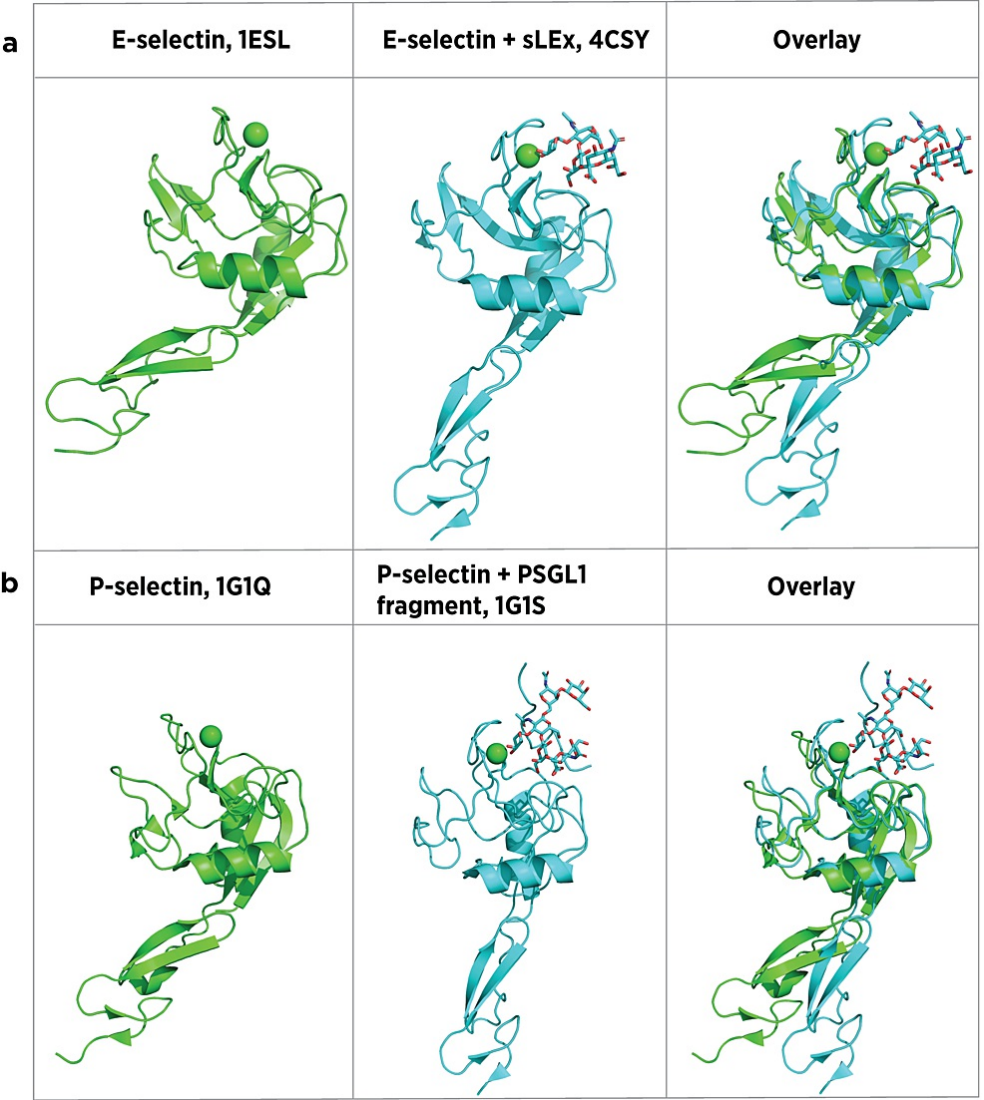
Bound and unbound selectin structures (a) Crystal structures of CRD and EGF-like domains of E-selectin unbound and bound to sLe^x^. (b) Crystal structures of P-selectin CRD and EGF-like domains unbound and bound to the PSGL-1 derived sulfoglycopeptide, SGP-3. RCSB Protein Data Bank reference numbers are shown. CRD: carbohydrate-recognition domain, EGF: epidermal growth factor, sLe^x^: sialyl Lewis^x^, PSGL-1: P-selectin glycoprotein ligand-1, RCSB: Research Collaboratory for Structural Bioinformatics Image credits: Dr. Drew Provan using Pymol

E-selectin and P-selectin CRD and EGF domain structures adopt a bent conformation in an unbound state [16]. Upon ligand binding, a conformational change to an extended state occurs via a pivot between the CRD and EGF domains (Figure 8) [16,109]. This conformational change is thought to form the basis of a "catch bond" that serves as a molecular brake to pull cells out of circulation against the shear force of venular blood flow [31,112]. Catch bonds strengthen with increasing applied force up to a point and then break with an increased force beyond that threshold [112]. This behavior contrasts with the behavior of slip bonds, which weaken with increasing applied force. Flow chamber measurements of neutrophil-selectin interactions display biphasic behavior that is flow rate dependent, indicating catch bond behavior [15,27,29]. Flow chamber studies were confirmed using atomic force microscopy. P-selectin/PSGL-1 bond lifetimes increase with up to about 30 pN of applied force and then decrease with forces > 30 pN [113]. Similarly, L-selectin showed catch bond behavior when interacting with PSGL-1 at shear < 50 pN [114]. E-selectin catch bonds were demonstrated using a bead capture assay [31].

**Figure 9:**
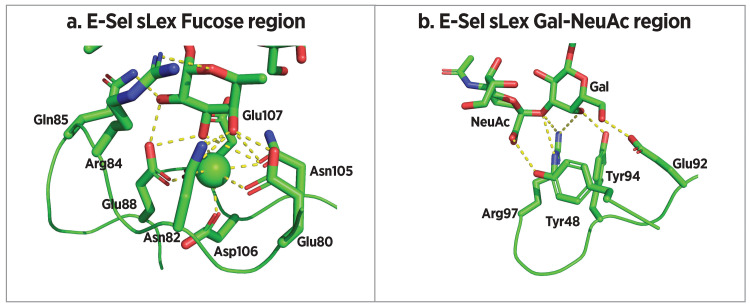
E-selectin bound to sLex (4CSY) (a) Interactions between E-selectin and the fucose residue of sLe^x^. (b) Interactions between E-selectin and the galactose and NeuAc residues of sLe^x^. sLe^x^: sialyl Lewis^x^ Image credits: Dr. Drew Provan using Pymol

**Figure 10:**
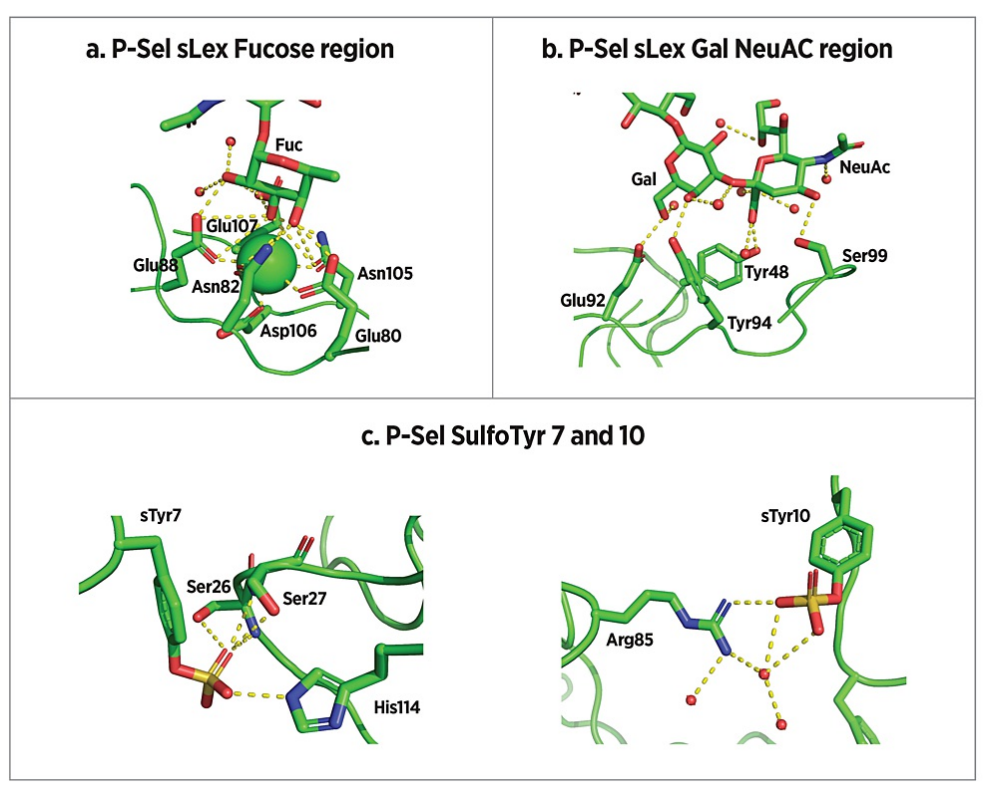
P-selectin bound to PSGL-1 derived glycosulfopeptide SGP-3 (a) Interactions between P-selectin and the fucose residue of SGP-3. (b) Interactions between P-selectin and SGP-3 galactose and NeuAc residues. (c) Interactions between P-selectin and SGP-3 sulfotyrosine residues 7 and 10. Image credits: Dr. Drew Provan using Pymol

Crystal structures of E-selectin and P-selectin bound to sLe^x^ indicate key binding interactions (Figure 9 and Figure 10) [16,109,110]. Knowledge of these interactions informs the rational design of selectin antagonists. Somers et al. [16] reported E-selectin (1ESL) and the same in which sLe^x^ was soaked into the 1ESL crystal (1GIT), demonstrating little change in protein conformation. By contrast, Preston et al. [109] found that E-selectin co-crystallization with sLe^x^ (4CSY) led to a change in angle between CRD and EGF domains from 120° in the unbound form reported by Somers et al. to 154° in the bound form. Prior binding of the E-selectin-sLe^x^ complex before co-crystallization suggests that 4CSY is the more physiologically relevant complex structure. Within co-crystal structure 4CSY, fucose 3- and 4-hydroxyl groups of sLe^x^ chelate the essential calcium ion that defines C-type lectins, and the fucose 2-hydroxyl group forms H-bonds with Gln85 and Glu88 (Figure 9a). On the sialic acid moiety, carboxylate carbonyl oxygen forms a hydrogen bond with Tyr48 while O-1 interacts with Arg97. Finally, galactose 3- and 4-OH groups also interact with Arg97, while the Gal O-6 hydroxyl forms a hydrogen bond with Glu92. The GlcNAc residue serves as a scaffold to maintain the location and orientation of these other binding elements but does not make direct contact with the protein (Figure 9b). Taken together, these structural interactions comprise the basis of E-selectin specificity, induce conversion from a bent to an extended conformation, and account for unique catch bond qualities.

Somers et al. [16] reported crystal structures of P-selectin CRD and EGF domains (1G1Q), as well as its co-crystal with synthetic glycosulfopeptide SGP-3 (1G1S) (Figure 8b). SGP-3 contains 19 N-terminal amino acids of PSGL-1, in which tyrosine residues at positions 5, 7, and 10 are sulfated (Figure 2b) and Thr16 is glycosylated with a sLe^x^-containing saccharide. P-selectin/sLe^x^ interactions are similar to those reported above for E-selectin/sLe^x^ interactions with the addition of a hydrogen bond between Ser99 and sialic acid O-4 (Figure 10a and Figure 10b). Sulfated tyrosines 7 and 10 make unique interactions with P-selectin. The sulfate of Tyr10 forms a hydrogen bond with Arg85, while that of Tyr7 forms hydrogen bonds with His114, Ser26, and Ser27 (Figure 10c).

In summary, selectin-ligand structural features yield several important conclusions. First, these interactions allow for bonds that are strong enough to pull circulating leukocytes out of circulation yet weak enough to enable cell rolling along the endothelial surface. Second, additional features beyond sLe^x^ alone that are recognized by P-selectin help explain the differential specificity of P-selectin versus E-selectin. Finally, knowledge of these interactions guides drug design.

Selectin antagonists tested in clinical trials

**Figure 11:**
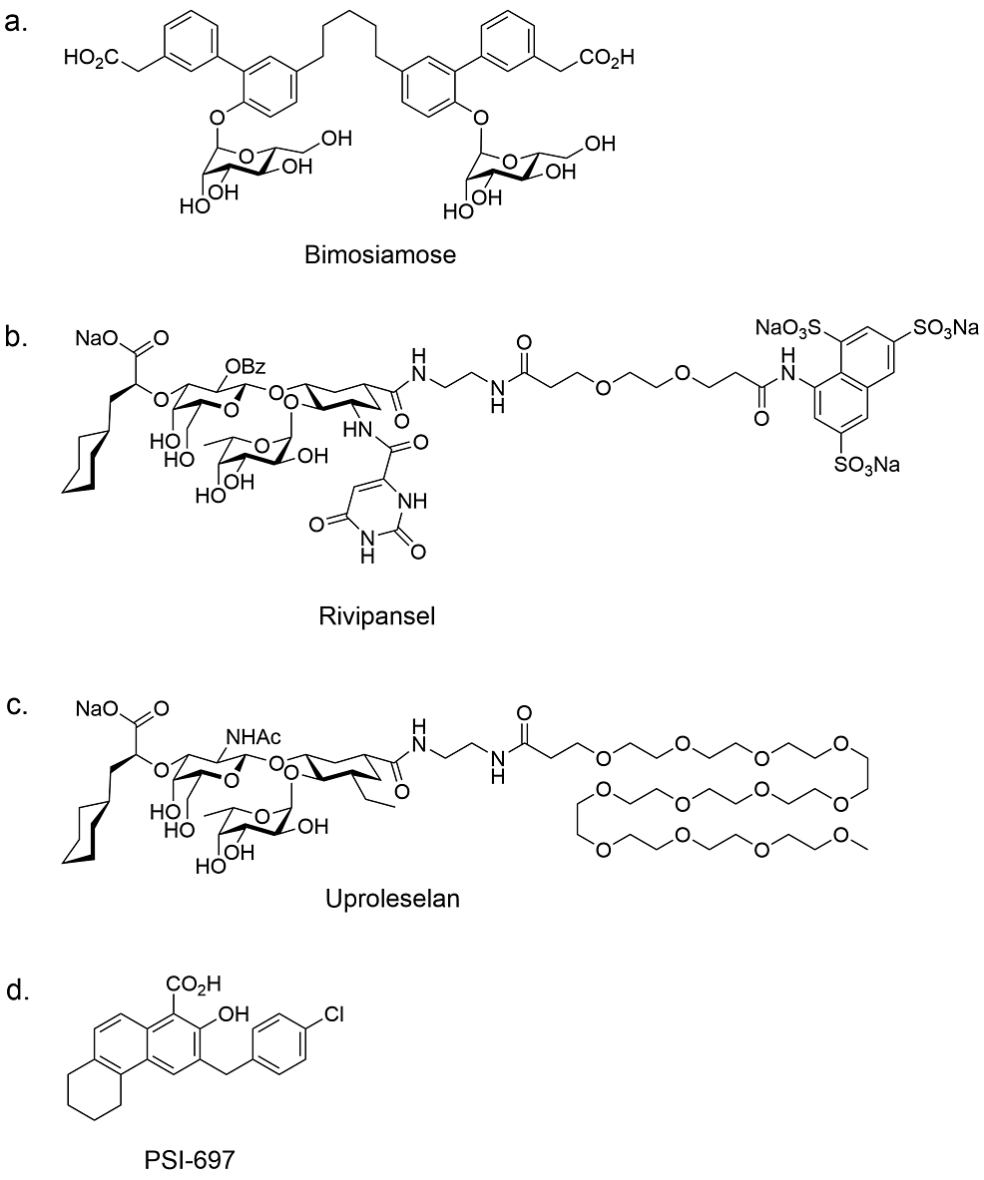
Structures of selectin antagonists that have been reported Image credits: Dr. Drew Provan using Adobe Illustrator

Numerous small molecule and antibody selectin antagonists have been reported with varying degrees of selectivity. This paper describes those that have advanced to human clinical trials. A brief summary of these agents is presented in Table 3, and the structures of those agents that have been reported are shown in Figure 11.

**Table 3:** Selectin antagonists tested clinically References: [115-121]

Sponsor	Structure type	Potency
Pan-selectin antagonists		
Cylexin	Small molecule	Not reported
Bimosiamose	Small molecule	E-selectin IC50 = 88 $\mu$M
		P-selectin IC50 = 20 $\mu$M
		L-selectin IC50 = 86 $\mu$M
Rivipansel	Small molecule	E-selectin IC50 = 4.3 $\mu$M
		P-selectin IC50 = 423 $\mu$M
		L-selectin IC50 = 337 $\mu$M
Sevuparin	Heparinoid polymer	E-selectin Kd = 19 $\mu$M
		P-selectin Kd = 0.038 $\mu$M
		L-selectin Kd = 0.95 $\mu$M
E-selectin-specific antagonists		
Uproleselan	Small molecule	E-selectin IC50 = 2.37 $\mu$M
		P-selectin IC50 > 10,000 $\mu$M
		L-selectin IC50 = 4,516 $\mu$M
GMI-1687	Small molecule	E-selectin IC50 = 0.16 $\mu$M
		P-selectin IC50 > 25,000 $\mu$M
		L-selectin IC50 > 7,000 $\mu$M
CDP850	mAb	E-selectin Kd = 1.6 nM
PF-07209326	mAb	E-selectin Kd = 68 nM
P-selectin-specific antagonists		
PSI-697	Small molecule	P-selectin IC50 = 125 $\mu$M
Crizanlizumab	mAb	P-selectin Kd = 5.9 nM
Inclacumab	mAb	P-selectin Kd = 9.9 nM

Pan-selectin Antagonists

Cylexin: The first selectin antagonist to reach human clinical trials was the carbohydrate ligand Cylexin (CI-1053), developed by Cytel Corporation. While the structure was not reported, publications and patent applications suggest it contains an embedded sLe^x^ [122-125]. Cylexin was assessed in a trial of patients with lung reperfusion injury during pulmonary thromboendoarterectomy. The study drug decreased lung injury but did not improve outcomes [126]. Also, in a trial of reperfusion injury after neonatal cardiopulmonary bypass surgery (NCT00226369), Cylexin showed a trend toward decreased 30-day mortality (https://ichgcp.net/clinical-trials-registry/NCT00226369).

Bimosiamose: Carboxy biaryl mannose dimer bimosiamose (TBC-1269) was discovered at Texas Biotechnology Corp. and developed by Revotar Pharmaceuticals (Figure 11a). Mannose moieties serve as surrogates for fucose in sLe^x/a^ while the carboxylate was designed to mimic sialic acid in sLe^x/a^ [127]. Bimosiamose is a pan-selectin antagonist having modest potency versus E-selectin, P-selectin, and L-selectin [115]. It attenuated allergen-induced late asthmatic reactions when administered by inhalation to asthmatic patients [128]. Also, bimosiamose attenuated airway inflammation in chronic obstructive pulmonary disease patients [129]. In a small trial of patients with plaque psoriasis, subcutaneous injection of bimosiamose reduced the psoriasis area and severity index [130]. No development has been reported since 2011.

Rivipansel: Rivipansel is a pan-selectin inhibitor invented by GlycoMimetics, Inc. and licensed to Pfizer (Figure 11b). Rivipansel mimics both sLe^x^ for selectin binding and sulfotyrosines for P-selectin and L-selectin binding. Since the GlcNAc of sLe^x^ was shown by crystallographic studies not to interact with the protein, it is replaced with a substituted cyclohexane ring. The sialic acid residue interacts with the protein via its carboxylate carbonyl group and its C-4 hydroxyl group. In rivipansel, the sialic acid residue is mimicked by a cyclohexyl lactic acid, which maintains the possibility of the carbonyl binding but loses the C-4 OH interaction. Sulfotyrosines are mimicked by a naphthalene trisulfonic acid. In ELISA testing of E-selectin, P-selectin, and L-selectin, rivipansel binds to all three selectins with the most potent activity against E-selectin [116].

The potential utility of rivipansel to treat vaso-occlusion was tested in a murine VOE model based on Berkeley sickle cell mice [131]. These mice express human $\alpha$-, $\gamma$-, and $\beta$-S-globin genes and not their murine counterparts [132]. They display major features of human SCD, including sickled RBCs, anemia, and multi-organ pathology. Colonies are difficult to maintain due to high perinatal mortality and low breeding efficiency. These difficulties were overcome by generating SCD chimeric mice through the transplantation of marrow cells from Berkeley sickle cell mice into lethally irradiated C57BL/6 mice. To model VOE, the endothelium was activated via injection of TNF-$\alpha$. After 110 minutes, a time sufficient to allow E-selectin and P-selectin expression and formation of occlusions, a 20 mg/kg rivipansel or phosphate-buffered saline (PBS) vehicle was administered. Rivipansel significantly decreased WBC adhesion to endothelium, decreased sRBC interactions with adherent WBCs, and increased blood flow rates relative to vehicle-treated controls [116].

Rivipansel advanced to clinical trials for VOE treatment in SCD patients. In a phase 2 clinical trial, it reduced the need for pain medication in sickle cell disease patients undergoing a VOE [133]. After rivipansel did not meet statistical significance in its pre-specified phase 3 primary analysis, post hoc analysis showed that early treatment led to significant, clinically meaningful decreases in opioid use and time to hospital discharge, providing proof of mechanism support for the potential utility of selectin antagonism as a therapeutic target in sickle cell VOE therapy [134].

Sevuparin: Sevuparin is a low-molecular-weight heparinoid developed by Modus Therapeutics. It has measurable binding activity to all three selectins via surface plasmon resonance assays, with the highest activity against P-selectin (Kd = 38 nM), followed by L-selectin (Kd = 950 nM) and then by E-selectin (Kd = 19 $\mu$M) [117]. Sevuparin was tested in a murine model of vaso-occlusion in which nude mice were treated with TNF-$\alpha$ to induce P-selectin and E-selectin expression. After four hours, labeled red blood cells from human SCD patients were administered. Sevuparin dose-dependently decreased adhesion of human red blood cell adhesion to vessel walls and normalized blood flow [117]. In a phase 2 clinical trial, it failed to decrease the median time to VOE resolution [135].

E-selectin-Specific Antagonists

**Figure 12:**
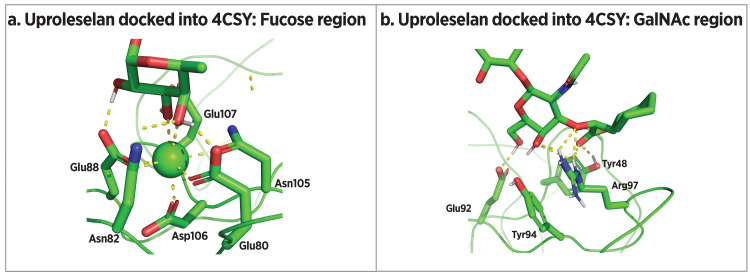
Docking of uproleselan into E-selectin crystal structure 4CSY (a) Predicted interactions in the fucose region of uproleselan. (b) Predicted interactions in the GalNAc-cyclohexylmethyl lactic acid region of uproleselan [109]. Image credits: Dr. Drew Provan using Pymol

Uproleselan: Uproleselan is a specific E-selectin antagonist developed by GlycoMimetics, Inc. (Figure 11c). Designed to mimic sLe^x^, uproleselan builds from rivipansel with the GlcNAc of sLe^x^ replaced by a substituted cyclohexane ring and the sialic acid replaced by a cyclohexyl lactic acid. The sulfotyrosine mimic of rivipansel is replaced by a C-12 polyethylene glycol (PEG) chain, both to remove activity against P-selectin and L-selectin and to improve pharmacokinetic properties (unpublished data). Docking uproleselan into an E-selectin crystal structure (4CSY reported by Preston et al. [109]) suggests that it makes similar interactions with the protein as sLe^x^ (Figure 12). The fucose O-3 and O-4 residues chelate calcium, while fucose O-2 is predicted to contact Glu88. The Gal O-6 hydroxyl is predicted to interact with Glu92, as is the case with sLe^x^. In addition, Tyr48 and Arg97 are both predicted to interact with Gal O-3 and the carbonyl of the cyclohexylmethyl lactic acid moiety. These interactions are similar to those between the galactose and sialic acid of sLe^x^.

Toxicology and healthy volunteer safety: Uproleselan demonstrates less than 10% plasma protein binding, is not metabolized by liver microsomes, and does not induce or inhibit CYP enzymes or P-glycoprotein [136]. It is neither mutagenic nor clastogenic. In both rodents and non-human primates, no observed adverse effects were seen at the highest dose level tested in each study. In four healthy volunteer studies, uproleselan demonstrated unremarkable toxicity approximating placebo and no changes in bleeding time or coagulation parameters [136]. Pharmacokinetics were linear and did not vary over time, comparing day 1 and day 5 dosing. A mass balance study confirmed negligible metabolism and complete urinary excretion.

Acute lung injury/COVID-19 infection: A phase 1 clinical trial assessed the safety, tolerability, and efficacy of uproleselan in patients with severe COVID-19 pneumonia requiring hospital admission for hypoxemia. Six patients received intravenous uproleselan (20 mg/kg BID on days 1-7, up to a maximum dose of 2,500 mg, or until hospital discharge). All patients were discharged to home, five without oxygen. None required mechanical ventilation or intubation, and no adverse events were reported (First Human Study to Assess the Safety of Selectin Inhibitor Uproleselan (GMI-1271) in Patients with COVID-19 Severe Pneumonia by Rocha OY, Sharma SB, Wong J, Sood S, Henke P, Hemmer MV, Magnani JL, Wakefield T, Napolitano LM, and Obi AT, American Venous Forum, February 22-25, 2023). Further studies on COVID-19 pneumonia and other acute lung injuries are warranted.

Human thromboembolic disease: Culmer et al. [137] used electrolysis to induce thrombi in an electrolytic inferior vena cava mouse model to study and compare the effects of uproleselan and low-molecular-weight heparin (LMWH) on venous thrombosis (VT). Two days later, the thrombus is excised and weighed. This model enables testing effects of anti-thrombotic agents under shear flow conditions. Uproleselan dose-dependently lowered thrombus weight and decreased inflammatory cell extravasation through the vein wall after both treatment and pre-treatment regimens. Importantly, uproleselan also significantly lowered tail vein bleeding times, compared to LMWH.

In a primate VT model, Myers et al. [138] evaluated uproleselan alone and in combination with LMWH. Following temporary iliac vein occlusion by balloon, baboons were treated with 25 mg/kg uproleselan subcutaneously, alone or combined with 1.5 mg/kg LMWH, once daily for 19 days. Vein recanalization was assessed by magnetic resonance venography and ultrasound. Vein recanalization was highest in the uproleselan alone group. Also, uproleselan alone decreased intimal thickening and intimal fibrosis.

Devata et al. [139] evaluated uproleselan in two patients with calf vein thrombosis. Both patients reported reduced pain and swelling following uproleselan administration. Duplex ultrasound of one patient demonstrated that the thrombosed posterior tibial vein remained occluded but that the length of the occlusion decreased, while in the other patient, the tibial vein was completely recanalized. In addition to improving vein wall recanalization, uproleselan decreased vein wall inflammation, intimal thickening, and fibrosis without the bleeding risk seen with other anticoagulants. Overall, these data provide proof of the mechanism that uproleselan is clinically active in VT therapy.

Uproleselan in hematologic malignancies: E-selectin antagonism has also been proposed as an approach to reduce chemoresistance of acute myeloid leukemia (AML) [140]. Uproleselan was evaluated in a phase 1/2 clinical trial as adjunctive therapy with chemotherapy in patients with relapsed/refractory AML [81] and is currently being tested in a phase 3 clinical trial as adjunctive therapy with chemotherapy in patients with relapsed/refractory AML (NCT03616470). E-selectin actions in hematopoiesis and uproleselan activity in hematologic cancers have been recently reviewed [136].

GMI-1687 (second-generation selective E-selectin antagonist): GMI-1687 is a highly potent, second-generation selective E-selectin antagonist developed by GlycoMimetics, Inc. In a surface plasmon resonance assay, it has a Kd of 2.4 nM (~3.8 ng/mL). It demonstrates high subcutaneous bioavailability in rodents and primates (unpublished data), as well as mechanistic activity in several animal models with inflammatory components.

GMI-1687 was assessed in two different models of acute SCD-related VOE [141]. In the first, three cohorts of nude mice were treated with TNF-$\alpha$ to activate endothelial E-selectin expression to capture circulating leukocytes. After two hours, mice were injected with rhodamine-labeled human red blood cells from donors with sickle cell disease (hSSRBC). After a further 30 minutes, subcutaneous doses of 0.04 mg/kg and 0.08 mg/kg dose-dependently decreased hSSRBC adhesion and percentage of occluded vessels while increasing the percentage with normal blood flow. In the related Townes model of severe human sickle cell disease [142], three cohorts of mice were treated with TNF-$\alpha$ to activate endothelium and initiate a VOE. After one hour, rhodamine 6G and phycoerythrin-labeled anti-mouse TER-119 antibodies were administered to label leukocytes and red blood cells, respectively. After a further 30 minutes, intravenous administration of GMI-1687 at 0.04 mg/kg and 0.08 mg/kg dose-dependently decreased cell adhesion and normalized blood flow. These data suggest that GMI-1687 has the potential to treat SCD VOE by early subcutaneous point-of-care dosing.

Human safety and pharmacokinetic properties of GMI-1687 were assessed in a single ascending dose study in healthy volunteers after subcutaneous doses of 3.3, 10, 20, 40, and 80 mg [143]. No dose-limiting toxicities, dose-related adverse events, or serious adverse events were observed. Also, GMI-1687 displayed linear pharmacokinetics with dose-dependent exposure. Phase 1 single ascending and multiple ascending dose studies in people living with SCD are planned.

CDP850 and PF-07209326 (anti-E-selectin antibodies): Several anti-E-selectin monoclonal antibodies have been developed. An anti-E-selectin antibody, CDP850, was developed at Celltech Therapeutics [118]. In vitro, it has a Kd of 1.6 nM and blocked leukocyte binding to activated endothelial cells. In a clinical trial of plaque psoriasis patients, CDP850 did not decrease severity scores [144].

Scientists at Pfizer developed an anti-E-selectin antibody, PF-07209326 [119], to be used to treat VOE in SCD patients. After phase 1 clinical testing, PF-07209326 was discontinued in August 2023 [145].

P-selectin-Specific Antagonists

PSI-697: The tetrahydro-benzoquinoline PSI-697 is a selective P-selectin inhibitor developed by scientists at Wyeth (now Pfizer) (Figure 11d) [120,146]. It displays modest potency in an assay measuring the binding inhibition of P-selectin CRD and EGF domains to immobilized PSGL-1 but failed to inhibit platelet-monocyte aggregates in human smokers [147]. In a small clinical trial using an ex vivo Badimon chamber model, PSI-697 reduced thrombus area [148]. No development has been reported since 2009.

Crizanlizumab and inclacumab (anti-P-selectin antibodies): Anti-P-selectin antibody crizanlizumab was developed by Selexys/Novartis for VOE prevention in SCD patients. Crizanlizumab decreased SCD VOE incidence by 45% in the phase 2 SUSTAIN study [149,150] and was approved by the Food and Drug Administration (FDA) in 2019 to reduce the frequency of VOE in adults and pediatric patients aged 16 years and older with sickle cell disease. It was also approved by the European Medicines Agency (EMA) in 2020 using the conditional marketing authorization (CMA) pathway. A second trial failed to replicate the initial findings, and EMA withdrew its CMA [151].

Another anti-P-selectin antibody, inclacumab, was discovered at Roche and initially developed to treat cardiovascular disorders [121,152]. It was reported to reduce myocardial damage during percutaneous intervention in myocardial infarction patients [153,154]. Inclacumab is now being studied in a Pfizer-sponsored phase 3 clinical trial to evaluate the VOE rate over 48 weeks (NCT05348915).

## Conclusions

In summary, E-selectin participates in the capture, rolling, and initiation of leukocyte firm adhesion to activated endothelium. Among selectins, only E-selectin connects early inflammatory leukocyte tethering/rolling to firm adhesion via effects on integrin affinity states. Also, E-selectin contributes to the pathology of diverse inflammatory disorders. Various selectin antagonists with varying selectivity for E-selectin have been developed and tested clinically, although none is yet approved for clinical use. A unifying biologic feature of E-selectin antagonism may be that in order to be effective, it must occur before firm adhesion is complete when the importance of E-selectin binding is eclipsed by subsequent high-affinity integrin binding.

Going forward, selective E-selectin antagonist uproleselan, administered intravenously, is now in phase 3 clinical trial as an adjunctive therapy for relapsed/refractory AML (NCT03616470). GMI-1687, a second-generation, more potent E-selectin antagonist, recently entered clinical testing as a subcutaneously delivered agent for the treatment of SCD VOE. If successful, GMI-1687 could allow for home administration at VOE onset. Finally, future identification of an orally bioavailable E-selectin antagonist may allow for treatment in the form of a pill, substantially expanding the potential clinical utility of E-selectin antagonism. In summary, therapeutic E-selectin antagonism has broad potential to address multiple diverse high unmet medical needs.
